# SCUBE1: a promising biomarker in renal cell cancer

**DOI:** 10.1590/S1677-5538.IBJU.2016.0316

**Published:** 2017

**Authors:** Ersagun Karagüzel, Ahmet Menteşe, İlke O.Kazaz, Selim Demir, Asim Örem, Ali Ertan Okatan, Diler Us Altay, Serap Özer Yaman

**Affiliations:** 1 Department of Urology, Karadeniz Technical University, Faculty of Medicine, Trabzon, Turkey;; 2 Program of Medical Laboratory Techniques, Karadeniz Technical University, Vocational School of Health Sciences, Trabzon, Turkey;; 3 Department of Nutrition and Dietetics, Karadeniz Technical University, Faculty of Health Sciences, Trabzon, Turkey;; 4 Department of Medical Biochemistry, Karadeniz Technical University, Faculty of Medicine, Trabzon, Turkey;; 5 Department of Chemistry and Chemical Processing Technology, Ordu University, Ulubey Vocational School, Ordu, Turkey

**Keywords:** Carcinoma, Renal Cell, Biomarkers

## Abstract

**Purpose:**

To investigate the efficacy of signal peptide-CUB-EGF domain-containing protein 1 (SCUBE-1) as a novel biomarker of renal tumors.

**Materials and Methods:**

48 individuals were included in the study. The patient group (Group-1) consisted of 23 subjects diagnosed with renal tumor, and the control group (Group-2) of 25 healthy individuals. Patients diagnosed with renal tumor received surgical treatment consisting of radical or partial nephrectomy. Blood specimens were collected following overnight fasting. Signal peptide-CUB-EGF domain-containing protein 1 (SCUBE-1), soluble urokinase plasminogen activator receptor (suPAR) and carbonic anhydrase IX (CA IX) levels were measured from plasma samples. Patients in groups 1 and 2 were compared in terms of these biochemical parameters.

**Results:**

The 23-member renal tumor group was made up of 17 (73.91%) male and 6 (26.08%) female patients with a mean age of 58.5±15.7 years (range 25 to 80). The 24-member healthy control group was made up of 16 (64%) male and 9 (36%) female subjects with a mean age of 52.4±9.12 years (range 40 to 67). Analysis revealed significant elevation in SCUBE-1 levels in the renal tumor group (p=0.005). No significant differences were detected between the groups with regard to CA IX or suPAR measurements (p=0.062 vs. p=0.176).

**Conclusions:**

SCUBE-1 appears to represent a promising biomarker in the diagnosis and follow-up of patients with renal tumor.

## INTRODUCTION

Renal cell carcinoma represents 2-3% of all adult malignancy neoplasms and is the most lethal urological cancer (1). Clear cell renal cell carcinoma (CCRCC) constitutes approximately 80% of renal cell carcinomas and follows a more aggressive course than papillary and chromophobe carcinoma, other pathological subtypes. Metastasis is present at diagnosis in approximate 1/3 patients, and there is a 30-40% risk of metastasis developing after surgery in subjects with localized disease at time of diagnosis (2). Radical or partial nephrectomy is the current gold standard in the treatment of localized disease.

Clinical, anatomical and histopathological criteria are used to determine prognosis of renal cancer. However, research aimed at identifying a biomarker that can be used in diagnosis, monitoring and determining tumors with a high risk of recurrence and that can show the need for early adjuvant and advanced treatment in metastatic patients is still ongoing.

Transmembrane carbonic anhydrase IX (CAIX) is one component of the carbonic anhydrase family. It is an efficient catalyst of the reversible hydration of carbon dioxide into bicarbonate and a proton. This permits tumor cells to preserve a neutral pH in the presence of an acidic microenvironment. Expression of CAIX does not occur in healthy kidney tissue. However, it does occur in the majority of CCRCCs. This occurs by way of HIF-1a overexpression caused by hypoxia and inactivation of the von Hippel-Landau gene (3). Some studies have described CA IX as a prognostic marker in patients with metastatic clear-cell renal cell carcinoma (mccRCC) (4). CAIX, one of the most studied biomarker in CCRCC, is considered promising.

Soluble urokinase plasminogen activator receptor (suPAR) is a glycosylphosphatidylinositol (GPI) membrane protein that binds to urokinase-type plasminogen activator receptor (uPAR) in soluble form (5) suPAR is produced by various types of cells, such as vascular endothelial cells, neutrophils and monocytes, and is thought to be associated with chronic inflammatory conditions (6). Studies have shown that suPAR is correlated with poor prognosis in some types of cancer (7, 8).

Signal peptide-CUB-EGF domain-containing protein 1 (SCUBE-1) is a cell surface glycoprotein. This novel biochemical marker is expressed and secreted in early embryogenesis and is present in platelets and endothelial cells (9). The SCUBE gene family contains three different isoforms, including SCUBE-1 (SCUBE 1-3) (10). SCUBE-1, a cell surface protein, has been investigated in various types of cancer and non-cancer diseases.

Based on the objective of developing a biomarker capable of use in renal tumors, we investigated SCUBE-1, a marker that has not previously been studied in patients with renal tumor. We compared SCUBE-1, a potentially novel marker, with CA IX and suPAR, previously investigated markers in renal tumors.

## MATERIAL AND METHODS

### Study population

Forty-eight individuals were included in the study, 23 patients diagnosed with renal tumor (Group-1) and a control group of 25 healthy subjects (Group-2). All members of both groups provided informed consent. The Karadeniz Technical University Medical Faculty Ethical Committee approved the study. All of the patients were evaluated clinically and they were also previously biochemical and radiologically investigated. Surgical treatment in the form of radical or partial nephrectomy was performed in all cases of diagnosed renal tumor.

### Blood samples

Blood samples were collected from patients following overnight fasting. These were taken from the peripheral vein and stored at 4ºC. Plasma specimens were obtained by centrifuging the blood samples at 3000rpm for 10 min. Plasma specimens were then stored at -80ºC until biochemical analysis.

### Biochemical measurements

#### Measurement of signal peptide-CUB-EGF domain-containing protein 1 (SCUBE-1) levels.

Levels of SCUBE-1 were determined using an enzyme-linked immunosorbent assay kit (Cusabio Biotech Co., Catalog No. CSB-E15005h, P.R. China) in line with the manufacturer’s instructions. The absorbance of samples was measured at 450nm using a VersaMax tunable microplate reader (designed by Molecular Devices in California, USA). The results were expressed as ng/mL. The minimum detectable level of human SCUBE-1 is generally lower than 0.16ng/mL.

#### Measurement of soluble urokinase plasminogen activator receptor (suPAR) levels.

Levels of suPAR were determined using an enzyme-linked immunosorbent assay kit (ViroGates A/S., Denmark) following the manufacturer’s protocols. The absorbance of samples was measured at 450nm using a VersaMax tunable microplate reader (designed by Molecular Devices in California, USA). The results were expressed as ng/mL. The estimated detection limit was 0.1ng/mL.

#### Measurement of carbonic anhydrase IX (CA IX) levels.

Serum levels of human CA IX were determined using an enzyme-linked immunosorbent assay kit (R&D systems, Catalog No. DCA 900, P.R. China) in line with the manufacturer’s protocols. The absorbance of samples was measured at 450nm using a VersaMax tunable microplate reader (Designed by Molecular Devices in California, USA). The results were expressed as pg/mL. The minimum detectable dose of human CA IX is generally lower than 2.28pg/mL.

## Statistical analysis

Statistical analyses were performed using computer software (SPSS version 13.0 software, Chicago, Illinois, USA). Data were expressed as mean±standard deviation. The Mann-Whitney U test and t-test were used for statistical analyses. Spearman correlation analysis was used to determine the correlation between biochemical parameters in the groups. Statistical signiﬁcance was set at p<0.05.

## RESULTS

Forty-eight patients were enrolled in the study. The renal tumor group consisted of 23 patients, 17 (73.91%) of whom were male and 6 (26.08%) female, with a mean age of 58.5±15.7 (range 25 to 80). The healthy control group consisted of 24 subjects, 16 (64%) male and 9 (36%) female, with a mean age of 52.4±9.12 (range 40 to 67). Tumors were removed by the methods of radical nephrectomy and partial nephrectomy (nephron sparing surgery) in 18 patients (78.3%) and 5 patients (21.7%), respectively. The pathological distribution of the tumors (pathological type, Fuhrman’s nuclear grade, pathological stage) in patients is shown in Table-1.

**Table 1:** The pathological distribution of the tumors in patients.

	n (%)
**Pathological type**	
Clear Cell RCC	17 (73.9)
Papillary RCC	4 (17.3)
Chromophobe RCC	2 (8.6)
**Fuhrman’s nuclear grade**	
Grade 1	7 (30.4)
Grade 2	11 (47.8)
Grade 3	3 (13.0)
Grade 4	2 (8.6)
**Pathological stage**	
pT1a	10 (43.4)
pT1b	7 (30.4)
pT2a	3 (13.0)
pT2b	2 (8.6)
pT3a	1 (4.3)

**RCC =** Renal cell cancer

Distribution of biochemical parameters in the two groups is shown in Table-2. Comparison of groups 1 and 2 revealed significantly elevated SCUBE-1 levels in the patients with renal tumor (p=0.005). No significant differences were observed between groups 1 and 2 in terms of CA IX or suPAR values (p=0.062 vs. p=0.176). There were also no significant differences between groups in terms of pathological type and stage and the Fuhrman’s grade (p>0.05).

**Table 2:** Comparison of biochemical parameters in the patient and control groups.

Biochemical parameters	Group 1 Patient group	Group 2 Control group	P value (p<0.05)
SCUBE1 (ng/mL)	14.80 ± 3.17	8.60 ± 5.22	0.005
CAIX (pg/mL)	59.06 ± 61.38	43.39 ± 61.73	0.176
suPAR (ng/mL)	7.54 ± 6.31	4.29 ± 5.23	0.062

**SCUBE-1 =** Signal peptide-CUB-EGF domain-containing protein 1; **CA IX =** Carbonic anhydraseIX; **suPAR =** Soluble urokinase plasminogen activator receptor

Spearman correlation analysis results of SCUBE-1, CAIX and suPAR in patient, control group and total sample is shown in Table-3. There was no correlation between biochemical parameters in patient, control group and total sample.

**Table 3:** Correlation analysis of biochemical parameters in patient, control group and total sample.

Biochemical parameters	Group 1 Patient group	Group 2 Control group	Total sample

	r - p	r - p	r - p
SCUBE-1 (ng/mL)- CAIX (pg/mL)	0.090 - 0.683	0.083 – 0.692	0.112 - 0.450
SCUBE-1 - suPAR (ng/mL)	0.152 – 0.487	0.173 – 0.408	0.131 – 0.375
CAIX (pg/mL) - suPAR (ng/mL)	0.058 – 0.792	0.115 – 0.585	0.153 – 0.299

**SCUBE-1 =** Signal peptide-CUB-EGF domain-containing protein 1; **CA IX =** Carbonic anhydraseIX; **suPAR =** Soluble urokinase plasminogen activator receptor

**r =** represents pearson coefficient of correlation; **p =** represents significance of coefficient of correlation

## DISCUSSION

Considerable advances have been made in recent years in the diagnosis of renal cancers, methods of treatment and prognosis. However, there is still a need for a marker with high sensitivity and specificity capable of use in the diagnosis and in determining prognosis of renal cancers.

Several studies have been performed with the aim of developing a biomarker with a high predictive value in renal tumors (11). CAIX is one biomarker that has been investigated for this purpose. CAIX was first tested as a predictive biomarker in a phase II study (SELECT trail), but the results were unsuccessful. CAIX was also investigated in mccRCC diagnosed patients using sorafenib, but no predictive or prognostic value was observed (12). Zhang et al. also concluded that CAIX is not an independent prognostic marker in patients diagnosed with CCRCC (13).

suPAR is a cell surface glycoprotein. Increased serum levels have been shown in several types of cancer. Elevated serum levels are closely correlated wıth poor prognosis (14, 15). In their study of patients with prostate cancer, Wach et al. revealed that high suPAR levels are a poor prognostic factor correlated with disease-specific survival (7). In a study of patients with gastrointestinal cancer, Zubkiewicz et al. suggested that suPAR is a significant prognostic marker in deciding on treatment in these cases (8).

SCUBE-1 is a member of the SCUBE gene family. Three different isoforms occur in mammals, SCUBE-1, -2 and -3. SCUBE-1 is a cell surface protein present in platelets and endothelial cells that is expressed and secreted in early embryogenesis (9). SCUBE-1 molecules are stored in alpha granules in inactive platelets. While thrombin is activated by platelets, SCUBE-1 is expressed on the platelet surface as a result of surface expression of the adhesion molecule P-selectin (9). It is released in the form of small, soluble particles incorporated into thrombus (10). In humans, it has robustly been shown in platelets and in fibrin-rich areas in organized thrombus (16).

SCUBE-1 has been investigated as a marker in non-cancer diseases (17-21). Türkmen et al. described SCUBE-1 as a potential marker capable of use in the early stage of acute mesenteric ischemia, in the specific diagnosis of pulmonary embolism and in the early diagnosis of acute ischemic stroke (17-19). SCUBE-1 has also been described as a valuable biomarker in determining severity and prognosis of disease in patients with Crimean-Congo hemorrhagic fever (20). Ozkan et al. determined high SCUBE-1 levels in patients with hypertension and suggested that SCUBE-1 may be an early biomarker of potential thrombotic complications occurring in association with hypertension (21).

Various studies have investigated and shown a close association between cancers and thrombosis (22-24). Some tumors trigger the coagulation cascade and procoagulant substances and initiate the inflammatory process. Procoagulant substances are released from tumor cells with the inflammatory process (25). One in vitro study determined a decrease in SCUBE-1 concentrations with interleukin-1-β and TNF-α therapies, a finding implicating SCUBE-1 in the inflammatory process (10). Expression of SCUBE-1 transcripts in prostate cancer stromal cells was encountered in a series analysis of prostate mesenchymal cell gene expressions (26). Mentese et al. concluded that SCUBE-1 is a useful marker in determining recurrences that may occur after treatment in patients with stomach cancer (27). Topcu et al. reported that SCUBE-1 can be effective in determining the risk of thrombosis and in screening patients to receive anti-thrombotic therapy as a marker of hypercoagulability in patients with breast cancer (28). In the light of these studies, SCUBE-1 may be of significant value as a biomarker in renal cancer, with widespread angiogenesis and thrombosis. We also determined significantly higher SCUBE-1 values in patients with renal cell cancer. But there was no correlation between SCUBE-1 values and the pathological parameters like type, grade and stage. On the other hand, when we investigated the distribution of renal tumor stages in patients, there was only one patient with grade T3a renal tumor. Almost all of the patients were diagnosed and treated in early stages of the disease in our patient group. This could be a significant advantage for SCUBE-1 as an early cancer detection biomarker considering the distribution of the patients in the group.

The most important limitation of this study is the low number of patients with renal tumor and control cases enrolled. Further multicenter studies with larger patient series are now needed on this subject.

## CONCLUSIONS

Research is continuing into different biomarkers in the diagnosis and prognosis and determination of response to treatment in renal tumors. In our study, SCUBE-1 levels were significantly elevated in cases of renal tumor. SCUBE-1 is a promising biomarker in the diagnosis and monitoring of patients with renal tumor, and further research with high number of cases is required.
